# Seroprevalence, Direct Detection and Risk Factors for *Toxoplasma gondii* Infection in Pigs in Serbia, and Influence of Biosecurity Measures

**DOI:** 10.3390/microorganisms10051069

**Published:** 2022-05-23

**Authors:** Nikola Betić, Nedjeljko Karabasil, Olgica Djurković-Djaković, Vladimir Ćirković, Branko Bobić, Ivana Branković Lazić, Vesna Djordjević, Ivana Klun

**Affiliations:** 1 Institute of Meat Hygiene and Technology, 11000 Belgrade, Serbia; nikola.betic@inmes.rs (N.B.); ivana.brankovic@inmes.rs (I.B.L.); vesna.djordjevic@inmes.rs (V.D.); 2 Department of Food Hygiene and Technology, Faculty of Veterinary Medicine, University of Belgrade, 11000 Belgrade, Serbia; nedja@vet.bg.ac.rs; 3 Centre of Excellence for Food- and Vector-Borne Zoonoses, Institute for Medical Research, University of Belgrade, 11129 Belgrade, Serbia; olgicadj@imi.bg.ac.rs (O.D.-D.); vladimir.cirkovic@imi.bg.ac.rs (V.Ć.); bobicb@imi.bg.ac.rs (B.B.)

**Keywords:** *Toxoplasma gondii*, pigs, seroprevalence, risk factors, farm biosecurity, mouse bioassay, direct detection, public health risk, Serbia

## Abstract

Consumption of *Toxoplasma gondii* contaminated pork is a major risk factor for human infection. We thus conducted a cross-sectional survey on the seroprevalence of *T. gondii* infection in a representative sample of slaughter pigs from throughout Serbia and examined the influence of farm biosecurity-related risk factors on infection. In addition, direct detection of the parasite (by mouse bioassay) or its DNA was performed in the hearts of a subset of seropositive sows. The overall seroprevalence in the sample of 825 pigs as determined by the modified agglutination test (MAT) was 16.5%. Older age and inadequate rodent control were independent infection risk factors for pigs. In a subset of 581 pigs with complete biosecurity-related data, in addition to older age, smallholders’ finishing type farms (as opposed to farrow-to-finish), multispecies farming, and origin from Western and Central and South-Eastern Serbia (vs. the Northern region), all increased the risk of infection, while the absence of disinfection boot-dips in front of each barn and Belgrade district origin (vs. the Northern region) were associated with a 62% and 75% lower risk of infection, respectively. Evidence of viable parasites was obtained in 13 (41.9%) of the 31 bioassayed sow hearts, of which by isolation of brain cysts in seven, by detection of *T. gondii* DNA in an additional four, and by serology in another two. Recovery of brain cysts mostly (5/7) from sows with a MAT titre of ≥1:100 indicates the risk for consumers. These results highlight the public health risk from pork consumption and point to mandatory use of professional rodent control services, abstaining from multispecies farming, keeping disinfection boot-dips clean and freshly refilled, as well as strict implementation of zoo-hygienic measures on smallholders’ farms as specific farm biosecurity measures needed for its reduction.

## 1. Introduction

*Toxoplasma gondii* is an apicomplexan protozoon that is capable of infecting all warm-blooded animals, including humans and livestock. The life cycle of *T. gondii* is complex and involves three stages infective for all hosts, tachyzoites, bradyzoites (in tissue cysts) and sporozoites (in oocysts). Sexual reproduction of *T. gondii* occurs in the enterocytes of members of the Felidae family, who are therefore the only definitive hosts, and ultimately results in the formation of environmentally resistant oocysts that are a source of infection for herbivores. Asexual reproduction results in the development of tissue cysts in various organs, known to persist in the muscles and other predilection sites for the life of the host [1]. Transmission of the infection via meat harbouring tissue cysts is extremely important from the food hygiene standpoint.

Toxoplasmosis is estimated to be the most prevalent parasitic infection globally [2], and is of continued interest in both human and veterinary medicine [3]. A report on the global importance of foodborne parasites ranked *T. gondii* in fourth place [4], while in Europe it has been ranked as high as second [5]. Consumption of raw or undercooked meat as a dominant transmission pathway and the key risk factor for human infection has been well established, and differences in infection prevalence were correlated with those in food preparation culture and tradition [6,7,8,9,10]. Pork is the most important source of infection for humans [11,12], especially since infected pigs have been proven to harbour viable tissue cysts throughout their productive lifetime [13]. Moreover, due to the absence of clinical symptoms in infected animals, as well as the lack of practical and cost-effective methods of detection of *T. gondii* tissue cysts in meat at slaughter [2], infected pigs and contaminated pork remain unrecognized.

Pig farming is dominant in the animal husbandry in European Union (EU), with almost 250 million heads slaughtered in 2021 [14] (EU, 2022). Data for 2020 show that the EU is only second to China and precedes the USA, with an annual production of pig meat of 23 million tonnes, which makes for more than a fifth of the total of the world’s pork production [15]. In Serbia, approximately 2.2 million pigs are slaughtered annually [16]. Therefore, understanding the epidemiology of *T. gondii* infection on pig farms and the role of particular on-farm risk factors is extremely important for disease control [17]. However, the last seroepizootiological study to encompass the whole of Serbia was performed 16 years ago [18].

The general purpose of this study was to determine the risk factors of *T. gondii* infection in slaughter pigs in Serbia. Because *T. gondii* prevalence is known to change temporally due to various factors, we assessed the current country-wide seroprevalence of *T. gondii* infection in pigs and analysed the influence of on-farm risk factors on the infection. The level of biosecurity on farms was determined via a standardized pork industry questionnaire that was used for the first time in this country. The possible effect of biosecurity measures on safeguarding public health was further assessed based on the level of direct detection of the parasite in the edible tissues (hearts) of slaughtered pigs.

## 2. Materials and Methods

### 2.1. Collection of Samples

The required sample size was calculated [19] based on the results of a previous study from Serbia [20] which also involved sampling of the slaughter pigs in several abattoirs, where the highest seroprevalence of *T. gondii* infection at any single abattoir amounted to 15.9%. For this expected seroprevalence, the minimum required sample size at the absolute precision of 5% for a 95% confidence interval (CI) was 206. Considering that the inter-abattoir variance for this study is unknown, and that in case of a higher variability the calculated sample sizes were bound to be conservative [19], the minimum required sample size was increased four-fold. Since the country is divided into 12 epizootiological units for formal epidemiological surveillance, the number of samples per unit was determined to adequately reflect the number of pigs raised for slaughter per unit.

Sample collection was undertaken from May 2017 to October 2019. All samples were collected at 15 abattoirs that slaughter pigs from throughout Serbia (excluding Kosovo and Metohija), where about three million pigs are bred [21]. These abattoirs included the four largest operations in the country, with an 85% share in the production in the 15 abattoirs, and that supply pork and pork products to all major food chains which cater to Serbian consumers; overall, the 15 abattoirs are responsible for more than 40% of the pork production in the country. The abattoirs were deemed representative since they slaughtered animals from all 12 epizootiological units, and they provided all the necessary logistics for sampling. At each abattoir visit, pigs from any epizootiological unit were randomly sampled, i.e., samples were collected from every fifth animal slaughtered. Blood samples were obtained during bleeding at the slaughter line directly into sterile collection tubes and stored in a portable cooler. The samples were transported to the National Reference Laboratory for Toxoplasmosis the same day, where centrifugation was performed for 17 min at 800× *g* at 4 °C. Separated sera were labelled and stored at −18 °C until serological testing.

Following the first round of sample collection, at three abattoirs with proportionally more seropositive pigs, additional samples were collected for direct detection of the parasite. Since the heart is an established predilection site for *T. gondii* tissue cysts [22], heart samples from sows were collected in parallel with the blood collection. Hearts were stored at refrigerator temperature (4–8 °C) until the serological test results were obtained (48–72 h). In case of positive serology, the heart was subjected to artificial digestion and part of the digested material was inoculated into mice, while part was used for *T. gondii* DNA testing. Of note, these sows were not included in the risk factor analysis so as not to over-represent the sample originating from these three abattoirs.

The study itself did not require ethical approval from the appropriate boards according to Serbian legislation (Animal Welfare Act, Official Gazette RS 41/2009), since the biological materials were collected from pigs on the slaughter line. The bioassay protocol was approved by the Veterinary Directorate Decision No. 323-07-02446/2014-05/1 of the Ministry of Agriculture and Environmental Protection of Serbia.

### 2.2. Collection of Epizootiological Data

The sampled animals were traced to the originating farms via veterinary health certificates and the origin of pigs was recorded; a detailed survey concerning biosecurity measures on farms and households was then performed. Standard questionnaires from the Australian Pork Industry Biosecurity Code Audit Checklist [23] were used, adapted to consider the specifics of swine husbandry and hygienic practices in Serbia. The pre-defined responses on the epizootiological data of interest are presented in Table 1. 

The survey was conducted by a researcher (NB) who either personally toured the farms or by means of electronic communication or telephone conversation with the practicing farm veterinarian or the farm manager. For some of the pigs originating from pig traders’ operations, questionnaires could not be completed; thus, two separate analyses of putative risk factors were performed, one for all sampled animals and one for animals with complete epizootiological data.

### 2.3. Serology

Pig sera were screened for the presence of *T. gondii*-specific IgG antibodies by the modified agglutination test (MAT) as described by Desmonts and Remington [24], using formalin-fixed tachyzoites of the *T. gondii* RH strain as antigen [25]. Sera were tested in serial twofold dilutions starting at 1:25 and titrated to the end point; all sera reactive at the 1:25 dilution (designated as cut-off) were considered positive. Mice sera (from bioassays) were also tested by MAT, but at a starting dilution of 1:20 was considered as cut-off. A positive (a laboratory standard prepared according to the WHO reference serum assessed at 1000 IU/mL) and a negative control (PBS buffer) were included in each test run.

### 2.4. Trypsin Digestion

Heart tissue samples were trimmed to remove any excess fat and connective tissue and cut into 2 × 2 cm cubes. A total of 200 g of tissue was weighed out and coarsely blended in a food processor. The minced tissue was digested with 0.25% porcine trypsin (T4674, Sigma-Aldrich, St. Louis, MO, USA) in sterile saline (final volume of 300 mL), supplemented with 200× penicillin-streptomycin solution (PAA Laboratories GmbH, Pasching, Austria), and 27 μg/mL amoxicillin (Hemofarm, Vršac, Serbia) [26]. The digestion was performed at 37 °C for 1.5 h with continuous stirring. Next, the suspension was filtered through sterile gauze; the flowthrough was collected and washed three times with sterile saline, in between centrifugations for 10 min at 1800× *g* at 4 °C. This resulted in a 3–10 mL pellet. To prevent carry-over contamination during processing, all instruments including knives, other utensils, and the food processor were thoroughly washed between samples with a detergent solution, followed by decontamination with a 10% hypochlorite solution and finally rinsed with distilled water.

### 2.5. Biological Assay

Digested heart tissue was bioassayed in mice according to our laboratory standard protocol [27]. The mice were adult females of the Swiss-Webster strain, weighing >20 g, purchased from the Animal Facility of the Military Medical Academy in Belgrade. A volume of 1.2 mL of digest mixed with 100 μL gentamicin solution (0.8 mg/mL) was inoculated intraperitoneally (i.p.) into two mice per heart. During all six weeks of the experiment, water and food were available ad libitum, and mice were observed daily for signs of clinical disease. Severely ill animals exhibiting symptoms such as severe lethargy, refusal to eat or hemiparesis were sacrificed by cervical dislocation to prevent excessive suffering. After six weeks, the surviving mice were euthanized, and a blood sample and the brain were harvested from each mouse. The serum was separated and used for serological testing, while a squash preparation was made from a part of the brain, and the rest was homogenized with 1 mL of saline and cysts counted in 100 μL of homogenate on a total of four slides under 200× magnification of a phase contrast microscope. A bioassay was considered positive if at least one *T. gondii* brain cyst was observed, or if parasite DNA was detected in the homogenate, or if specific IgG antibodies were detected in the serum of at least one mouse.

### 2.6. DNA Extraction and Real-Time Polymerase Chain Reaction (qPCR)

DNA was extracted from a volume of 100 μL of heart digests, or of homogenized murine brain tissue, using the GeneJET Genomic DNA Purification Kit (Thermo Fisher Scientific, Waltham, MA, USA), as described previously [26].

Detection of *T. gondii* DNA in heart digests and brain homogenates was performed using qPCR, targeting the 529 bp repetitive element (Gene Bank accession number AF146527) [28]. Briefly, the PCR reaction was performed in a final volume of a 20 μL mixture comprising 10 μL of Maxima Probe/ROX qPCR Master Mix (2X) (Thermo Fisher Scientific, Waltham, MA, USA), 0.25 mM of each primer (HO1, forward-AGA GAC ACC GGA ATG CGA TCT; HO2, reverse-CCC TCT TCT CCA CTC TTC AAT TCT), 0.10 mM of TaqMan HOFT probe (FAM-ACG CTT TCC TCG TGG TGA TGG CG-TAMRA; Invitrogen, Life Technologies, Carlsbad, CA, USA), 0.015 U/μL of UNG, 25 mM MgCl_2_ and nuclease-free water, with addition of 3 μL of template i.e., extracted DNA. Amplification and detection was performed with the StepOnePlus™ real-time PCR system (Applied Biosystems, Waltham, MA, USA), using a programme consisting of 2 min at 50 °C for UNG pre-treatment, 5 min at 95 °C for initial denaturation, followed by 45 cycles of 15 s at 95 °C for denaturation and of 60 s at 60 °C each for annealing and extension.

Each PCR run included a negative and a positive extraction control, a negative PCR control (a DNA extract previously found to be negative), and a positive PCR control (a DNA extract previously found to be positive). A sample was declared positive if at least one of the duplicates was positive, i.e., having a Ct < 40 cycles. The limit of detection was determined to be 10 *T. gondii* tachyzoites per mL of sample.

### 2.7. Statistical Analysis

For all seroprevalence values obtained in the study, 95% CIs were calculated. The differences in seroprevalence between variable categories were determined by Chi-square analysis. For dichotomous as well as dummy variables, the reference category was the one with the highest frequency of observations, except for the variable ‘rodent control’, where categories were arranged according to the expected effects of the applied rodent control method. Univariate logistic regression was performed, and variables significant at the *p* ≤ 0.15 level were tested for collinearity and included in the multivariate models to analyse the influence of examined factors as independent categorical variables on *T. gondii* seroprevalence; the overall fit of the models was tested by Hosmer-Lemeshow goodness-of-fit statistics. Final models were obtained by the forward or backward stepwise method, as appropriate for the best fit. The results are presented as adjusted odds ratios (OR) with 95% CIs. Comparison of the specific antibody level in sows with the results of the bioassays and/or qPCR of heart digests was also analysed by logistic regression. Agreement between these two assays was interpreted according to the Cohen’s kappa values [29].

The level of significance was 5%. All statistics were performed using the SPSS version 11.5 statistical package (SPSS Inc., Chicago, IL, USA).

## 3. Results

### 3.1. T. gondii Seroprevalence and Risk Factors

**Figure 1:**
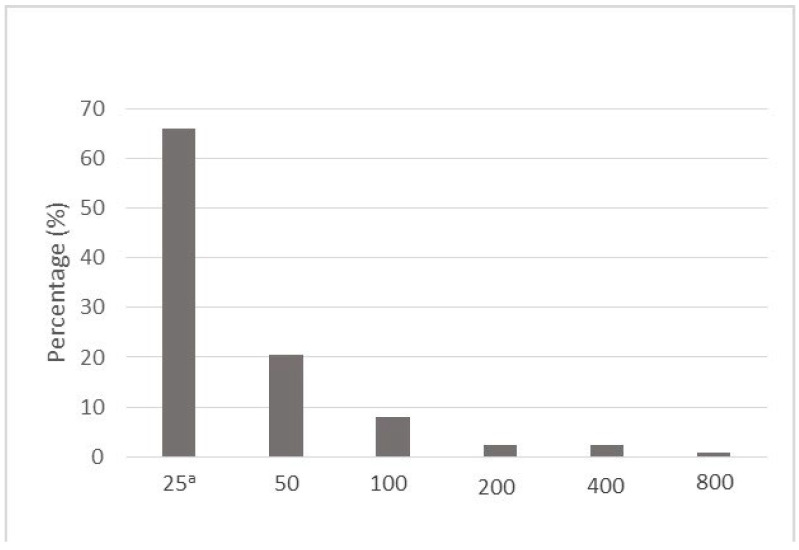
Distribution of *Toxoplasma gondii* antibody levels in seropositive slaughter pigs in Serbia. ^a^ Reciprocal of titre.

The overall seroprevalence in the series of 825 examined pigs was 16.5%, with specific antibody levels ranging from 1:25 to 1:800 (Figure 1). More than 80% of the pigs had low specific antibody titres of ≤1:50, while only one animal had a titre of 1:800.

**Figure 2:**
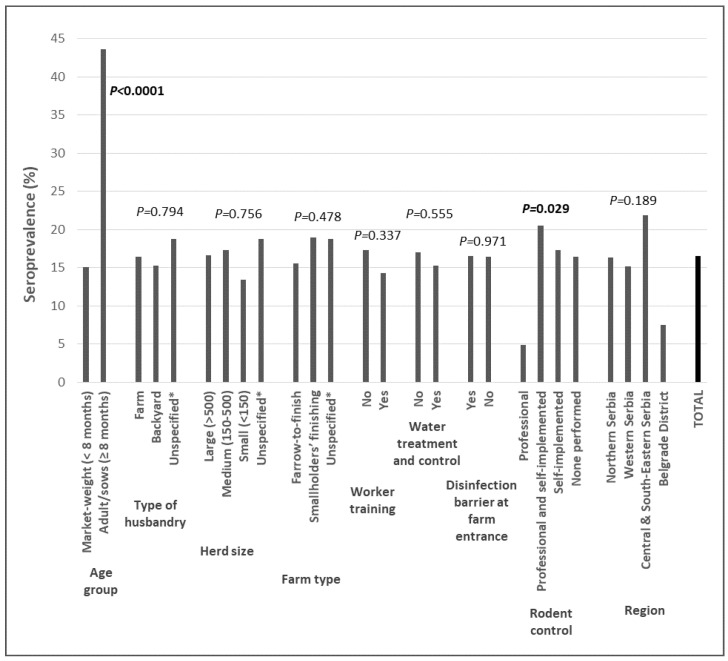
*Toxoplasma gondii* seroprevalence in all sampled pigs (N = 825) according to independent variable categories. *p* values for univariate logistic regression analysis (values ≤ 0.15 in bold). * pigs from pig traders’ operations.

The seroprevalence was, expectedly, significantly higher in sows (43.6%) than in market-weight pigs (15.1%). Also, *T. gondii* seroprevalence varied according to type of rodent control, being significantly lower on farms with professional rodent control as compared to all other modes of implementation (Figure 2, Table S1).

Age group and rodent control were thus included in the multivariate logistic model, which showed that both were independently associated with *T. gondii* infection. Adult pigs (sows) had a more than four-fold greater likelihood of being infected compared to market-weight pigs. As for rodent control, compared with professionally implemented programmes, other types increased the chances of pigs being infected by 3.3–4.5-fold. (Table 2).

**Figure 3:**
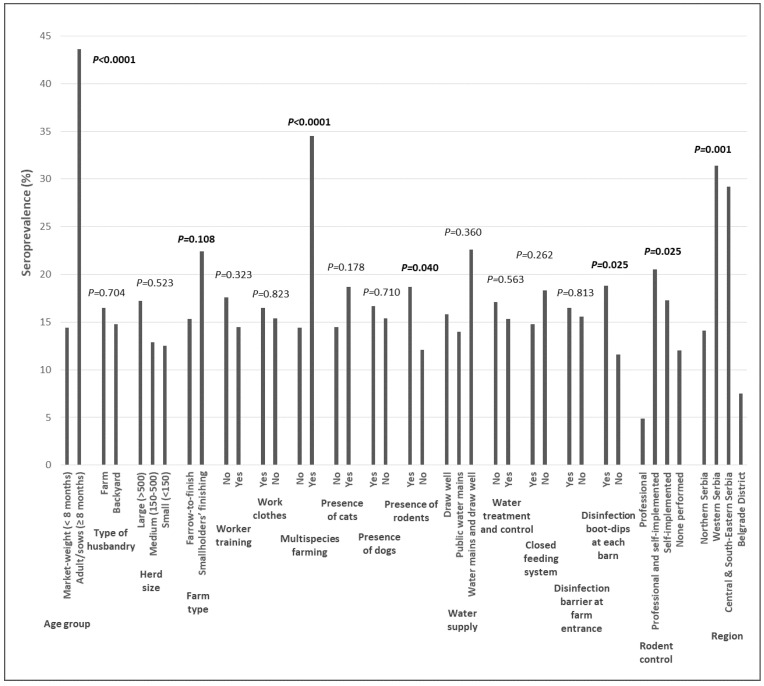
*Toxoplasma gondii* seroprevalence in pigs with complete biosecurity questionnaires (N = 581), according to independent variable categories. *p* values for univariate logistic regression analysis (values ≤ 0.15 in bold).

We next analysed pigs from farms for which fully completed biosecurity questionnaires were obtained, comprising a total of 581 pigs (70.4% of the total sample), in which the seroprevalence was 16.35% (95% CI = 13.4–19.6%). In this subgroup, univariate analysis showed that age group, farm type, multispecies farming, the presence of rodents, disinfection boot-dips at each barn, rodent control, and region were all associated with *T. gondii* seroprevalence in individual pigs (Figure 3, Table S2). These variables were included in multivariate analysis, with the backward stepwise method having the best model fit.

The final model showed that age group, farm type, multispecies farming, disinfection boot-dips in front of each barn as well as region were independently associated with *T. gondii* infection. Adult pigs had a more than 14-fold greater likelihood to be infected compared to market-weight pigs. The pigs from farms where other animal species were raised as well had a three-fold greater risk compared to those from farms where pigs were the sole animal raised. The absence of barn disinfection boot-dips was associated with a 61.5% lower risk of infection while the risk of infection for pigs from smallholders’ finishing type facilities was increased 4.5-fold compared to farrow-to-finish farms. Pigs from Western and Central & South-Eastern Serbia were more likely to be infected (by four- and two-fold, respectively) than pigs from Northern Serbia, while the Belgrade District origin had a protective effect of 75% compared to the Northern region (Table 3).

*T. gondii* infection seroprevalence was also analysed per farm as unit of analysis. Out of a total of 55 farms that fully completed the questionnaires, almost two thirds had at least one seropositive animal, with an overall seroprevalence of 61.8%. Univariate analysis of putative risk factors showed that, however, none of the variables were associated (*p* > 0.15) with *T. gondii* seroprevalence (results not shown).

### 3.2. Direct Detection of T. gondii and Viability Assessment

Blood and hearts were sampled from 68 sows at three abattoirs. A total of 33 sows were seropositive at a titre of ≥1:25, and their heart tissue tested for *T. gondii* DNA and as bioassayed. Two bioassay experiments were lost to the study due to lethal bacterial infections of the mice, therefore 31 bioassays were finalized, and the results are presented in Table 4. Seven (22.6%) heart digests were positive for *T. gondii* DNA. Interestingly, brain cysts were recovered from seven bioassays (22.6%) with a substantial overall agreement of 87.1% (Cohen’s kappa = 0.631) while, expectedly, more bioassays (11, 35.5%) were positive for *T. gondii* DNA in mouse brains. According to mice serology, 29% of the bioassays were positive (9/31, of which two were positive by serology alone). Cumulatively, evidence for the presence of viable *T. gondii* in the inoculated heart tissue was obtained in a total of 41.9% (13/31) of bioassays.

There was no influence of the sow MAT titre on the isolation success, nor on the detection of *T. gondii* DNA in the heart digests, as analysed by logistic regression. Nevertheless, most brain cyst isolations (5/7) were from sows with a titre of 1:100 or higher, while no cysts were detected in the brains of mice inoculated with heart digests from 13 sows with a MAT titre of 1:25. In one of these 13 bioassays, however, both the digest and the mouse brain were qPCR positive; in one, *T. gondii* DNA was detected only in the mouse brain, and one was only positive by serology.

## 4. Discussion

This study has shown an overall seroprevalence of *T. gondii* infection in slaughter pigs in Serbia of 16.5%, with the infection almost three-fold as prevalent in sows (43.6%) than in market-weight pigs (15.1%). Although the prevalence of infection can vary with time, the results per category are quite similar to those from a first seroepizootiological study that covered the entire territory of Serbia carried out 16 years ago, which showed a seroprevalence 40.9% in sows and 15.2% in fatteners [18]. Another study by Klun et al. [20] which specifically included the population of pigs on the slaughter line in three slaughterhouses in the Belgrade area, similarly showed that *T. gondii* was more common in sows (30%) than in pigs younger than eight months (8.3%), while the lower prevalence was attributed to the study area characterized by highly industrialized farming. A more recent study that only included market-weight pigs from a total of four large commercial farms and several smallholdings in the northern Serbian region of Vojvodina showed a higher prevalence of 17% [26], but which was due to an outbreak of *T. gondii* infection at one of the farms.

The seroprevalence determined in this study is in the range of values reported for some of the major pig rearing countries in the world; for instance, the seroprevalence in China is between 19 and 30%, USA 15–36%, Brazil 14–27%, Spain 13–26% and Germany 8–11% [30], and almost identical to the infection prevalence in parts of another southern European country—in Central (16.3%) and Southern Italy (16.1%) [31,32].

Risk factors for *T. gondii* infection for the total of 825 pigs were age and mode of rodent control. On the other hand, for the 581 pigs with complete biosecurity epidemiological data where a more precise risk factor analysis was possible, age group, farm type, multispecies farming, and disinfection boot-dips in front of each barn as well as region were all shown to be independently associated with *T. gondii* infection. Animal age is a well-known risk factor, shown in many studies, from Serbia [18,20] and elsewhere [31,33,34,35,36,37]. The seroprevalence of *T. gondii* infection in sows is consistently shown to be higher than in fattening pigs [38].

The study corroborated the significance of adequate rodent control; the absence of rodent control has been shown to be a major risk factor for infection [31]. The apparently paradoxical result of inefficient self-implemented as well as combined modes of rodent control may perhaps be explained by farmers either not using the rodenticides according to the manufacturer’s instructions or using a rodenticide antagonistic to the one used by professional rodent control services. Additionally, although the implementation of rodent control exclusively by professional pest control companies resulted in the lowest *T. gondii* prevalence on farms, this may also be the effect of a high level of biosecurity and stricter adherence to zoohygienic measures implemented on those farms (or reflects other factors not covered by the questionnaire). Moreover, farms that acknowledged the absence of organised rodent control measures often reported the use of cats as a means of rodent control, which may explain a higher seroprevalence on those farms, since the presence of cats is a risk factor for *T. gondii* infection in farmed pigs [36] as contaminants of stored feed, for instance.

Furthermore, the infection risk was 4.5-fold greater for pigs on smallholders’ finishing than on farrow-to-finish farms, which was shown in many other studies as well [18,31,32]. This is likely due to better control of husbandry conditions and production practices in farrow-to-finish farms, as well as to the absence of introducing replacement pigs from outside sources, while, conversely, finishing farms always depend on outside replacements.

Although only the presence of poultry was shown to be a risk factor for infection of pigs [31], multispecies farming is generally considered as an indication of low intensity farming and its specifics, such as more opportunities for feed, water, and soil contamination with oocysts, as well as contact with rodents [36], which have all been shown to increase the risk of *T. gondii* infection [17]. In the present study, an apparently lower zoohygienic status of such farms as compared to those that exclusively keep pigs was indeed observed during farm touring.

Proper implementation of stricter sanitary procedures is expected, and has been shown [39] to contribute to a further reduction of *T. gondii* infection. Surprisingly, in this study disinfection boot-dips at each barn seemingly did not serve their purpose and were in fact an infection risk factor. However, a close look during farm visits showed that at most of the farms these boot-dips were not topped up with fresh disinfectant; some were even dried out and may be an ideal spot for potential accumulation and dispersion of oocysts by workers. Indeed, low levels of staff hygiene was shown to be a risk factor [40], while proper maintenance of farm facilities had a protective effect [41]. Some studies have shown, however, that neither biosecurity measures, staff movement restrictions, nor the wearing of specialised boots or clothes had any influence on the prevalence of infection [34,41,42]. 

The study showed that infection risk was higher for pigs from Western, and Central and South-Eastern Serbia compared to the Northern (reference) region, which may be explained by the difference in husbandry, because the first two regions have very few large industrial farms and therefore biosecurity measures are generally at a lower level than in the Northern region. Conversely, the only farms in the Belgrade District are the highly industrialised ones, with adherence to strict biosecurity standards, obviously accounting for the lower risk of *T. gondii* infection compared to the Northern region in this and previous research [20].

No risk factors for *T. gondii* infection were identified when farms were taken as units of analysis. This may be surprising since analysis at farm level is preferable to the one at the individual animal level [43]. A possible explanation for the reduced level of association may lie in the overdispersion effect due to higher between-farm variability in the response variable [44].

Almost half of the bioassay experiments where naive mice were inoculated with digested sow heart tissue showed evidence of viable parasites. A substantial agreement was obtained between bioassay results and detection of *T. gondii* DNA in heart digests. The six cases of disagreement where the bioassays were positive, but no DNA was detected in the digests may be attributed to a small tissue volume for DNA extraction. The single discrepancy in the other direction, with a DNA positive heart digest and a negative bioassay, indicates the absence of viable cysts in the inoculum (suggesting a very low parasite burden in the heart, or questioning cyst viability), or that infection in mice could not be established even if they were, as is known to occur with some isolates [45]; this bioassay was additionally hampered by the demise of the second mouse from *T. gondii*-unrelated causes (*E. coli* infection in the sampled pigs or sample contamination).

We next analysed the relationship between specific antibody level and parasite isolation. Specific antibody levels in pigs from the present study varied from low (1:25, in two thirds of pigs) to moderately high (1:800, only one animal). Conversely, in our 2006 study [18], titres of ≥1:1600, suggestive of acute infection, were detected in a number of animals, with the highest titre being 1:12,800. No correlation of specific antibody titre with parasite isolation was found in this study (thus actually repeating the result of our 2011 study [20]); the fact that most (5/7) parasite isolations originated from sows with titres of 1:100 or higher suggests that this titre cut-off may be indicative of a higher risk for consumers. This issue was assessed by Herrero et al. [41], who showed that a titre of ≥ 1:80 is a good indication of the presence of viable cysts in meat.

The high rate of isolation success indicates a high public health risk, since, as stated above, the dominant mode of infection with *T. gondii* is via consumption of raw or undercooked meat, or inadequately processed meat products [38,46]. In fact, (a) as each slaughtered pig produces at least 300 meal servings [47]; (b) as pork is frequently mixed with meat from other animals in different meat products [46,48]; and (c), as sow meat is frequently used in products that undergo insufficient heat treatment or curing, the estimated seroprevalence and viability assessment in this study may point to a considerable number of meals potentially contaminated with viable cysts reaching the consumers in Serbia every year. This is especially important since pork makes up to 50% of the share of all meat types consumed in Serbia [18]. Even if a great share of contaminated meat usually undergoes post-harvest treatments that inactivate tissue cysts (especially cooking or other thermal processing), still a significant amount of contaminated meat is consumed inadequately processed, or improperly cured, which implies a considerable public health risk.

## 5. Conclusions

In conclusion, since the prevalence of *T. gondii* infection in pigs has been suggested to be a convenient indicator of the risk for human toxoplasmosis due to pork consumption [49], monitoring of the farm seroprevalence status and strict adherence to biosecurity measures can be a good start in mitigating the risk for consumers. Specific farm-level measures geared to the achievement of this goal as indicated by the results of this study in slaughter pigs in Serbia should include implementation of professionally organised rodent control programmes, boot disinfection prior to entrance in each housing barn, avoidance of multispecies farming especially at farms which cater to the meat industry, and raising the biosecurity level at smallholders’ finishing type farms.

## Figures and Tables

**Table 1:** Categories of data collected on plausible epizootiological risk factors for *Toxoplasma gondii* infection in pigs in Serbia.

Factor	Categories/Responses
Age group	Market-weight (<eight months); Adult/sows (≥eight months)
Type of husbandry	Farm; Backyard
Herd size	Large (>500); Medium (150–500); Small (<150)
Farm type	Farrow-to-finish; Smallholders’ finishing
Worker training	Yes; No
Work clothes	Yes; No
Multispecies farming	Yes; No
Presence of cats	Yes; No
Presence of dogs	Yes; No
Presence of rodents	Yes; No
Water supply	Draw well; Public water mains; Water mains and draw well
Water treatment and control	Yes; No
Closed feeding system	Yes; No
Disinfection barrier at farm entrance	Yes; No
Disinfection boot-dips at each barn	Yes; No
Rodent control	Professional; Professional and self-implemented; Self-implemented; None performed
Region	Northern Serbia; Western Serbia; Central & South-Eastern Serbia; Belgrade District

**Table 2:** Risk factors for *Toxoplasma gondii* infection in all sampled pigs (N = 825) in Serbia. Final multivariate model; individual pigs as units of analysis.

Factor	Adjusted OR	95% CI	*p* Value
**Age group**			
Market-weight (<eight months)	1.00		
Adult/sows (≥eight months)	4.197	2.104–8.374	<0.0001
**Rodent control**			
Professional	1.00		0.040
Professional and self-implemented	4.550	1.564–13.237	0.005
Self-implemented	3.304	1.144–9.543	0.027
None performed	3.813	1.327–10.957	0.013

**Table 3:** Risk factors for *Toxoplasma gondii* infection in pigs (N = 581, with complete biosecurity questionnaires only) in Serbia. Final multivariate model; individual pigs as units of analysis.

Factor	Adjusted OR	95% CI	*p* Value
**Age group**			
Market-weight (<eight months)	1.00		
Adult/sows (≥eight months)	14.247	6.258–32.438	<0.0001
**Farm type**			
Farrow-to-finish	1.00		
Smallholders’ finishing	4.508	2.160–9.406	<0.0001
**Multispecies farming**			
No	1.00		
Yes	3.092	1.480–6.459	0.003
**Disinfection boot-dips at each barn**			
Yes	1.00		
No	0.385	0.197–0.752	0.005
**Region**			
Northern Serbia	1.00		0.001
Western Serbia	4.014	1.777–9.070	0.001
Central & South-Eastern Serbia	2.264	1.051–4.877	0.037
Belgrade District	0.250	0.067–0.935	0.039

**Table 4:** Direct detection of *Toxoplasma gondii* from heart tissue of seropositive pigs. Outcome of bioassays of heart digests from 31 seropositive sows sampled at three abattoirs in Serbia.

Pig ID	MAT Titre (Pig Sera)	Real-Time PCR (Pig Heart Digest)	Mice with Cysts/Mice Examined (n) (Cyst Count)	Seropositive Mice/Mice Examined (n) (MAT Titre)	Real-Time PCR (Mouse Brain)
S1	1:25	-	0/2	0/2	0/2
S5	1:50	-	0/2	0/2	0/2
S10	1:25	-	0/2	0/2	0/2
S14	1:100	+	1/1 ^a^ (820/mL)	1/1 ^a^ (1:20,480)	2/2 ^†^
S16	1:50	-	1/2 (30/mL)	1/2 (1:40,960)	1/2
S19	1:50	-	0/2	0/2	0/2
S23	1:25	-	0/2	0/2	0/2
S24	1:100	-	0/2	0/2	1/2
S27	1:25	-	0/2	0/2	0/2
S29	1:50	-	0/2	0/2	0/2
S30	1:100	-	2/2 (30/mL; 10/mL)	2/2 (1:5120; 1:2560)	2/2
S33	1:25	+	0/0 ^b^	0/0 ^b^	1/1 ^b,†^
S34	1:25	-	0/2	1/2 (1:40)	0/2
S35	1:100	-	0/2	1/2 (1:20)	0/2
S36	1:200	+	0/1 ^c^	0/1 ^c^	0/1 ^c^
S37	1:400	+	1/1 ^d^ (<10/mL *)	1/1 ^d^ (1:40,960)	1/1 ^d^
S44	1:25	-	0/2	0/2	0/2
S46	1:50	+	1/1 ^e^ (20/mL)	1/1 ^e^ (1:20,480)	1/1 ^e^
S47	1:25	-	0/2	0/2	0/2
S48	1:25	-	0/2	0/2	0/2
S49	1:800	-	0/2	0/2	0/2
S50	1:25	-	0/2	0/2	0/2
S52	1:25	-	0/2	0/2	0/2
S53	1:50	-	0/2	0/2	0/2
S54	1:25	-	0/2	0/2	0/2
S58	1:100	-	0/2	0/2	0/2
S60	1:25	-	0/2	0/2	1/2
S64	1:50	-	0/2	0/2	0/2
S65	1:400	-	0/2	0/2	1/2
S66	1:100	+	1/2 (30/mL)	2/2 (1:5120; 1:5120)	2/2
S68	1:100	+	1/2 (10/mL)	2/2 (1:2560; 1:5120)	2/2

^a^ One mouse died 14 days post-infection (DPI) (^†^ qPCR: liver, heart). ^b^ One mouse died 10 DPI (not examined), one was sacrificed 14 DPI (^†^ qPCR: heart, lungs, liver). ^c^ One mouse died three DPI (Enterotoxigenic *Escherichia coli* isolated from peritoneal exudate (pex)). ^d^ One mouse died two DPI (ETEC in pex). ^e^ One mouse died 13 DPI. * Cysts detected in squash preparations only.

## Data Availability

Datasets available on demand. The data presented in this study are available in supplementary material.

## Supplementary Materials

The following supporting information can be downloaded at: https://www.mdpi.com/article/10.3390/microorganisms10051069/s1, Supplementary Table S1. *Toxoplasma gondii*-specific antibodies in all sampled pigs (N = 825) according to independent variables. Results of univariate logistic regression analysis; individual pigs as units of analysis. N—number examined, CI—confidence interval, OR—odds ratio; * pigs from pig traders’ operations. Table S2. *Toxoplasma gondii*-specific antibodies in pigs (N = 581, with complete biosecurity questionnaires only) according to independent variables. Results of univariate logistic regression analysis; individual pigs as units of analysis. N—number examined, CI—confidence interval, OR—odds ratio.
