# Supplementary material for: Seroprevalence, Direct Detection and Risk Factors for Toxoplasma gondii Infection in Pigs in Serbia, and Influence of Biosecurity Measures
Source: Microorganisms. 2022 May 23;10(5):1069. doi: 10.3390/microorganisms10051069 (PMC9146795; doi:10.3390/microorganisms10051069)
Supplement: Supplementary file 1 [file microorganisms-10-01069-s001.zip › microorganisms-1705371-supplementary.pdf]

Table S1. *Toxoplasma gondii*-specific antibodies in all sampled pigs (N = 825) according to independent variables. Results of univariate logistic regression analysis; individual pigs as units of analysis. N - number examined, CI - confidence interval, OR - odds ratio; *pigs from pig traders' operations

Factor	N	Prevalence (%)	95% CI	OR	95% CI	P value
**Age group**						**<0.0001**
Market-weight (< 8 months)	786	15.1	12.7-17.8			
Adult/sows (≥ 8 months)	39	43.6	27.8-60.4	4.331	2.233-8.399	
**Type of husbandry**						0.794
Farm	616	16.4	13.6-19.6			
Backyard	124	15.3	9.5-22.9	0.923	0.541-1.573	
Unspecified*	85	18.8	11.2-28.8	1.182	0.659-2.121	
**Herd size**						0.756
Large (>500)	511	16.6	13.5-20.1			
Medium (150-500)	110	17.3	10.7-25.6	1.046	0.606-1.807	
Small (<150)	119	13.4	7.9-20.9	0.779	0.438-1.385	
Unspecified*	85	18.8	11.2-28.8	1.162	0.643-2.100	
**Farm type**						0.478
Farrow-to-finish	576	15.5	13.6-18.7			
Smallholders' finishing	164	18.9	13.2-25.7	1.275	0.812-2.003	
Unspecified*	85	18.8	11.2-28.8	1.269	0.704-2.286	
**Worker training**						0.337
No	597	17.3	14.3-20.5			
Yes	228	14.3	10.2-19.7	0.822	0.530-1.242	
**Water treatment and control**						0.555
No	577	17.0	14.0-20.3			
Yes	248	15.3	11.1-20.4	0.884	0.588-1.330	
**Disinfection barrier at farm entrance**						0.971
Yes	472	16.5	13.3-20.2			
No	353	16.4	12.7-20.7	1.007	0.694-1.460	
**Rodent control**						**0.029**
Professional	82	4.9	1.3-12.0			
Professional and self-implemented	190	20.5	15.0-27.0	5.036	1.737-14.605	
Self-implemented	284	17.3	13.0-22.2	4.066	1.422-11.629	
None performed	269	16.4	12.1-21.3	3.813	1.327-10.957	
**Region**						0.189
Northern Serbia	520	16.3	13.3-19.8			
Western Serbia	151	15.2	9.9-22.0	0.920	0.557-1.518	
Central & South-Eastern Serbia	114	21.9	14.7-30.6	1.438	0.871-2.372	
Belgrade District	40	7.5	1.6-20.4	0.415	0.125-1.377	
**TOTAL**	**825**	**16.5**	**14.0-19.0**			

Table S2. *Toxoplasma gondii*-specific antibodies in pigs (N = 581, with complete biosecurity questionnaires only) according to independent variables. Results of univariate logistic regression analysis; individual pigs as units of analysis. N - number examined, CI - confidence interval, OR - odds ratio

Factor	N	Prevalence (%)	95% CI	OR	95% CI	P value
**Age group**						**<0.0001**
Market-weight (< 8 months)	542	14.4	11.5-17.6			
Adult/sows (≥ 8 months)	39	43.6	27.8-60.4	4.579	2.336-9.045	
**Type of husbandry**						0.704
Farm	526	16.5	13.7-20.0			
Backyard	55	14.8	6.5-26.7	0.859	0.392-1.881	

**Herd size**						0.523
Large (>500)	471	17.2	13.9-20.9			
Medium (150-500)	70	12.9	6.0-23.0	0.710	0.339-1.488	
Small (<150)	40	12.5	4.2-26.8	0.688	0.261-1.809	
**Farm type**						**0.108**
Farrow-to-finish	496	15.3	12.3-18.8			
Smallholders’ finishing	85	22.4	14.0-23.7	1.591	0.904-2.801	
**Worker training**						0.323
No	353	17.6	13.7-21.9			
Yes	228	14.5	10.2-19.7	0.794	0.502-1.258	
**Work clothes**						0.823
Yes	516	16.5	13.4-20.0			
No	65	15.4	7.6-26.5	0.922	0.452-1.880	
**Multispecies farming**						**<0.0001**
No	526	14.4	11.6-17.7			
Yes	55	34.5	22.2-48.6	3.125	1.704-5.732	
**Presence of cats**						0.178
No	330	14.5	10.9-18.8			
Yes	251	18.7	14.1-24.1	1.354	0.871-2.103	
**Presence of dogs**						0.710
Yes	419	16.7	10.2-21.9			
No	162	15.4	13.3-20.6	0.910	0.553-1.496	
**Presence of rodents**						**0.040**
Yes	374	18.7	14.9-23.0			
No	207	12.1	8.0-17.3	0.597	0.365-0.976	
**Water supply**						0.360
Draw well	469	15.8	12.6-19.4			
Public water mains	50	14.0	5.8-26.7	0.869	0.376-2.006	
Water mains and draw well	62	22.6	12.9-35.0	1.557	0.817-2.967	
**Water treatment and control**						0.563
No	333	17.1	13.2-21.6			
Yes	248	15.3	11.1-20.4	0.876	0.560-1.371	
**Closed feeding system**						0.262
Yes	330	14.8	11.2-19.1			
No	251	18.3	13.7-23.7	1.287	0.828-2.000	
**Disinfection barrier at farm entrance**						0.813
Yes	472	16.5	13.3-20.2			
No	109	15.6	9.4-23.8	0.933	0.527-1.653	
**Disinfection boot-dips at each barn**						**0.025**
Yes	382	18.8	15.0-23.1			
No	199	11.6	7.5-16.8	0.563	0.340-0.932	
**Rodent control**						**0.025**
Professional	82	4.9	1.3-12.0			
Professional and self-implemented	190	20.5	15.0-27.0	5.036	1.737-14.605	
Self-implemented	284	17.3	13.0-22.2	4.066	1.422-11.629	
None performed	25	12.0	2.5-31.2	2.659	0.553-12.781	
**Region**						**0.001**
Northern Serbia	441	14.1	10.9-17.7			
Western Serbia	35	31.4	16.7-49.1	2.802	1.307-6.006	
Central & South-Eastern Serbia	65	29.2	18.6-41.8	2.525	1.388-4.592	
Belgrade District	40	7.5	1.6-20.4	0.496	0.148-1.657	
